# Fabrication of Chitosan-Based Network Polysaccharide Nanogels

**DOI:** 10.3390/molecules27238384

**Published:** 2022-12-01

**Authors:** Aina Nakamichi, Jun-ichi Kadokawa

**Affiliations:** Graduate School of Science and Engineering, Kagoshima University, 1-21-40 Korimoto, Kagoshima 890-0065, Japan

**Keywords:** chemical crosslinking, functional maltooligosaccharide, nanogel, network polysaccharide, water-soluble chitosan

## Abstract

In this study, we developed a method to fabricate chitosan-based network polysaccharides via the condensation between amino groups in water-soluble chitosan (WSCS) and a carboxylate-terminated maltooligosaccharide crosslinker. We previously reported on the fabrication of network-polysaccharide-based macroscopic hydrogels via the chemical crosslinking of water-soluble chitin (WSCh) with the crosslinker. Because the molecular weight of the WSCS was much smaller than that of the WSCh, in the present investigation, the chemical crosslinking of the WSCS with the crosslinker was observed at the nanoscale upon the condensation between amino and carboxylate groups in the presence of a condensing agent, 1-ethyl-3-(3-dimethylaminopropyl)carbodiimide hydrochloride, and *N*-hydroxysuccinimide, affording nano-sized chitosan-based network polysaccharides. The occurrence of the crosslinking via the formation of amido linkages was supported by the IR analysis and ^1^H NMR measurements after the dissolution via acid hydrolysis in DCl/D_2_O. The products formed nanogels, whose sizes depended on the amino/carboxylate feed ratio. The nanoscale morphology and size of the products were evaluated via scanning electron microscopy, dynamic light scattering analyses, and transition electron microscopy. In the present study, we successfully developed the method to fabricate nanogel materials based on network polysaccharide structures, which can practically be applied as new polysaccharide-based 3D bionanomaterials.

## 1. Introduction

Natural polysaccharides, which consist of monosaccharide units linked through glycosidic bonds, are a major class of biopolymers and are identified as the most abundant organic resources on Earth [1,2]. Due to their controlled specific structures, they have been used for the fabrication of bio-based nanomaterials, such as nanoparticles, nanofibers, and nanogels, via the regular assembly of polymeric chains [3,4,5,6]. For example, polysaccharide-based nanoscopic hydrogels (nanogels) have been prepared via the construction of nanoscale network structures through the chemical or physical crosslinking of polysaccharide chains [7]. Nanogels are submicron-sized hydrogels that are formed via the chemical or physical crosslinking of polymeric chains to afford a three-dimensional (3D) network with water retention properties and no solubility in aqueous media [8]. Chitosan (CS) is one of the most widely used polysaccharide sources for nanogel preparations [9,10]. CS is prepared via the deacetylation of chitin, which is a natural aminopolysaccharide composed of β(1→4)-linked repeating *N*-acetyl-d-glucosamine units that are abundantly present as exoskeletons in crustaceans and insects [11]. CS-based nanogels have previously been fabricated via the chemical crosslinking of amino groups at the C-2 position with other moieties such as genipin, an iridoid glucoside extracted from the fruit of *Gardenia jasminoides* [12,13,14]. Physical crosslinking was achieved through the self-assembly of hydrophobically modified CS in water to produce nanogels [15,16,17].

To fabricate polymeric network matrices comprising saccharide components via chemical crosslinking, we successfully synthesized a maltooligosaccharide (α(1→4)-oligoglucan) crosslinker with carboxylate groups at both ends (carboxylate-terminated maltooligosaccharides, GlcA-Glc*_n_*-COONa) [18]. GlcA-Glc*_n_*-COONa was synthesized using thermostable glucan phosphorylase (GP, isolated from the thermophilic bacterium *Aquifex aeolicus* VF5)-catalyzed enzymatic α-glucuronylation using α-d-glucuronic acid 1-phosphate (GlcA-1-P) as a glycosyl donor and a maltooligosaccharide with a carboxylate group (Glc*_6_*-GlcCOONa) as a glycosyl acceptor (Figure S1) [19]; the latter was prepared using chemical oxidation at the reducing end of the maltoheptaose (Glc_7_) using sodium hypoiodite (I_2_/NaOH) [20]. The enzymatic α-glucuronylation is based on the fact that GP shows weak specificity for the recognition of glycosyl donors and catalyzes enzymatic glycosylations using non-native sugar 1-phosphate glycosyl donors as the analog substrate of the native α-d-glucose 1-phosphate (Glc-1-P) [21]. Subsequently, the terminal carboxylate groups in the resulting GlcA-Glc*_n_*-COONa were used for chemical crosslinking with amino groups in water-soluble chitin (WSCh) via condensation in the presence of a condensing agent in water to produce network chitins composed entirely of saccharide chains; WSCh is basically chitin with an *N*-acetylation degree of ca. 50%, which specifically shows water solubility [22]. The resulting network chitins formed hydrogels in the reaction mixture, and the hydrogels could be easily converted into films via pressing.

**Figure 1:**
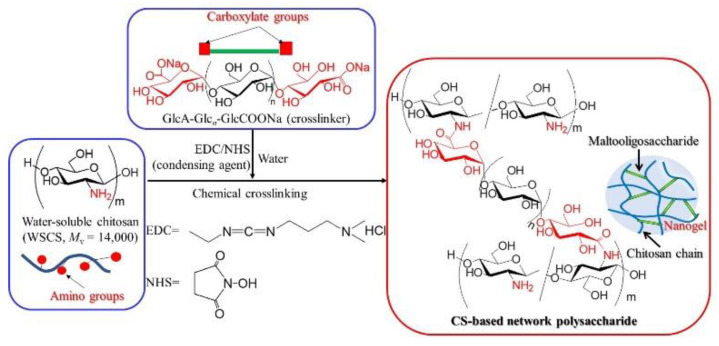
Chemical crosslinking of WSCS with GlcA-Glc*_n_*-COONa in the presence of a condensing agent (EDC/NHS) in water to produce a CS-based network polysaccharide.

In this study, we fabricated network polysaccharides on a smaller scale (nanogels), using a chemical crosslinking approach similar to the one described above, from GlcA-Glc*_n_*-COONa and water-soluble chitosan (WSCS) (Figure 1). WSCS and WSCh are completely different aminopolysaccharides. WSCS is a water-soluble, relatively low-molecular weight CS (approximately 1.0–5.0 × 10^4^) that is prepared via the complete deacetylation of chitin under alkaline conditions and with subsequent oxidative depolymerization via the treatment with hydrogen peroxide [23,24]. The chemical crosslinking to fabricate the nanogels was induced by the condensation of the amino groups in the WSCS with the terminal carboxylate groups in the GlcA-Glc*_n_*-COONa in the presence of a condensing agent in water. The nanoscale morphology and size of the products were evaluated using scanning electron microscopy (SEM), transition electron microscopy (TEM), and dynamic light scattering (DLS) analyses. Consequently, in this study, we developed the efficient method to fabricate nanogels based on network polysaccharide structures. As the existing nanogels are entirely composed of saccharide chains, their practical application as new polysaccharide-based 3D bionanomaterials can be expected.

## 2. Results and Discussion

We first prepared the GlcA-Glc*_n_*-COONa and WSCS according to procedures found in the literature [18,25]. The thermostable GP-catalyzed enzymatic α-glucuronylation of Glc*_6_*-GlcCOONa using GlcA-1-P was performed to introduce a GlcA residue at the non-reducing end and obtain GlcA-Glc*_n_*-COONa (Figure S1). The complete deacetylation of chitin under alkaline conditions, followed by oxidative depolymerization using hydrogen peroxide, afforded the WSCS. The viscosity rates of the products were measured to estimate the viscosity average molecular weight (*M*_v_) of the resulting WSCS according to the literature method [26], while the *M*_v_ was determined to be 14,000.

The chemical crosslinking of WSCS with GlcA-Glc*_n_*-COONa was carried out in the presence of a condensing agent (EDC and NHS, with the number of moles being equivalent to that of the COONa group) in water at room temperature for 7 h in a similar manner to that used for GlcA-Glc*_n_*-COONa/WSCh [18] to produce CS-based network polysaccharides (Figure 1). Different feed ratios of NH_2_ to COONa (1:0.04–1:0.12, runs 1–5 in Table 1) were examined to investigate the effect on the morphology and size of the products. Macroscopic hydrogels were not obtained in all reaction mixtures, unlike in the case of the crosslinked GlcA-Glc*_n_*-COONa/WSCh. Because we assumed that network polysaccharides at small scales, e.g., at the nanoscale, were produced, the reaction mixtures were poured into methanol to isolate the products. As the precipitates were obtained using this procedure, the products isolated as methanol-insoluble fractions were subjected to the following characterization procedures. The IR spectrum of the isolated product (run 4; Figure S2b) showed a new carbonyl absorption peak at 1640 cm^−1^, assignable to the amido linkage compared with that of WSCS (Figure S2a), indicating the occurrence of a condensation reaction between the NH_2_ and COONa groups.

**Figure 2:**
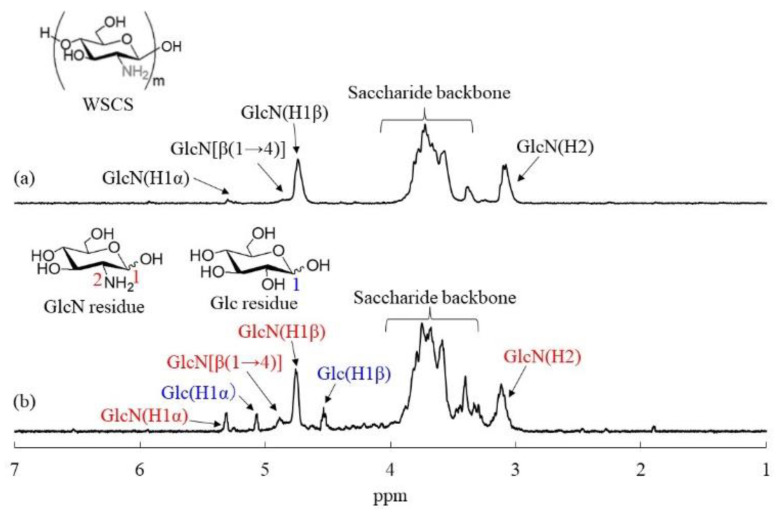
^1^H NMR spectra of (**a**) WSCS and (**b**) CS-based network polysaccharide (run 4) in DCl/D_2_O.

The structures of the network polysaccharides were further confirmed by ^1^H NMR measurements after the dissolution of the products via acid hydrolysis in DCl/D_2_O, as previously reported for the structural analysis of chitin derivatives [18,27,28]. The ^1^H NMR spectrum of the sample obtained after the dissolution of the product in run 4 in DCl/D_2_O (Figure 2b) showed anomeric signals not only in the glucosamine (GlcN) residues derived from the WSCS, but also in the Glc residues derived from the GlcA-Glc*_n_*-COONa, supporting the polysaccharide network structure; the anomeric signals in the Glc residues were not detected in the ^1^H NMR spectrum of the WSCS in the DCl/D_2_O (Figure 2a). The unit ratios, which were calculated from the integrated ratios of the anomeric signals (H1) at δ 4.54 and 5.07 (β and α, respectively) from the Glc residues to the H2 signals at δ 3.09 from the GlcN residues, increased according to the COONa/NH_2_ feed ratios (Table 1).

**Figure 3:**
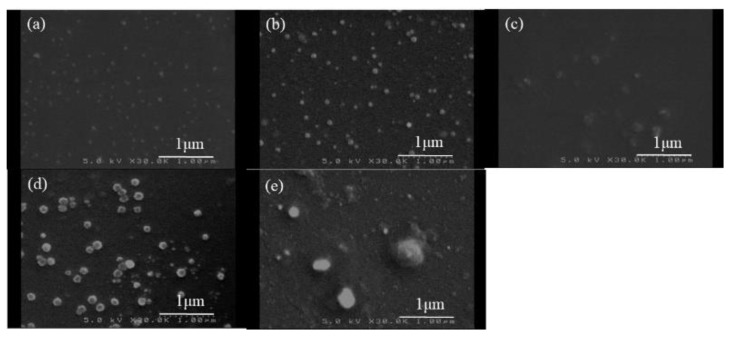
SEM images of CS-based network polysaccharides: (**a**–**e**) runs 1–5.

**Figure 4:**
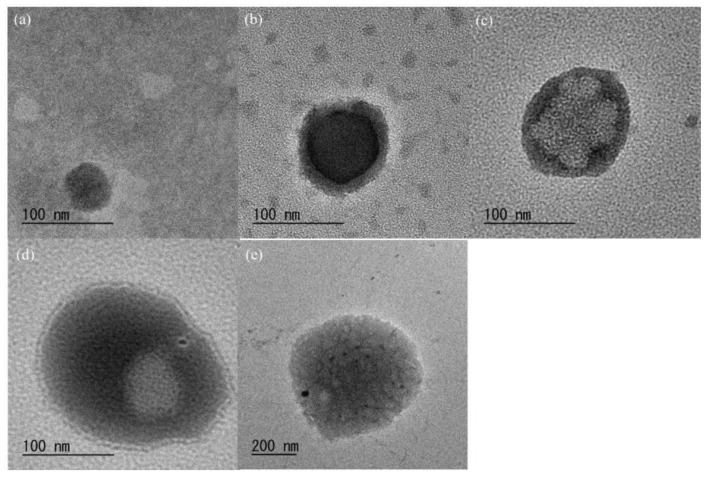
TEM images of CS-based network polysaccharides: (**a**–**e**) runs 1–5.

SEM measurements were performed to investigate the morphology of the CS-based network polysaccharides at the nanoscale level. The SEM images of the spin-coated samples from the aqueous dispersion of the products (Figure 3) show the particle morphology. The average particle diameters, which were calculated based on 50 objects for each SEM image to be 44–242 nm, increased with the increasing Glc/GlcN unit ratio (Table 1). This indicated that crosslinking of the CS chains with GlcA-Glc*_n_*-COONa occurred more frequently and complied with the COONa/NH_2_ feed ratios to produce larger particles. As listed in Table 1, furthermore, relatively small standard deviation values were evaluated based on the SEM images. The DLS profiles of the aqueous dispersion of the products (5.0 mg/10 mL) showed monomodal profiles at the submicron scale (Figure S3). The results also indicated an increase in particle size according to the Glc/GlcN unit ratios; however, these were larger than those estimated from the SEM images (Table 1). The different particle sizes obtained from the SEM and DLS analyses were attributable to the sample conditions (dry or wet) used in these measurements. The TEM images of the products (Figure 4) show a hollow morphology with some dots inside the particles, suggesting a network structure [29]. From the above morphological analysis, we concluded that the CS-based network polysaccharide nanogels were formed by the chemical crosslinking of the WSCS with the GlcA-Glc*_n_*-COONa in water.

## 3. Materials and Methods

### 3.1. Materials

The thermostable GP from *Aquifex aeolicus* VF5 was kindly provided by Dr. Takeshi Takaha (Sanwa Starch Co., LTD., Nara, Japan) [30]. The chitin powder from the crab shells was purchased from FUJIFILM Wako Pure Chemical Corporation (Osaka, Japan). The fully deacetylated chitosan was prepared via the deacetylation of chitin according to the literature procedure [31]. The GlcA-1-P was prepared via the TEMPO-mediated oxidation of Glc-1-P according to the literature procedure [32]. The Glc_7_ was prepared via the selective cleavage of a glycosidic bond of β-cyclodextrin under acidic conditions [33]. All other reagents and solvents were used as received from commercial sources.

### 3.2. Synthesis of GlcA-Glc_n_-GlcCOONa

A methanolic solution of I_2_ (3.62 g/24.4 mL) was added to an aqueous solution of Glc_7_ (5.70 g, 4.90 mmol/10 mL) and the mixture was stirred at 40 °C for 2 h. After adding a dropwise methanolic solution of NaOH (2.66 g/75.5 mL), the resulting mixture was stirred at 40 °C for 1 h. The precipitate was isolated via decantation and dried under reduced pressure at room temperature for 10 h. The product was dissolved in water (40 mL) and the solution was treated with activated carbon at room temperature for 1 h with stirring. After the activated carbon was filtered out, the filtrate was concentrated and lyophilized to give a crude Glc*_6_*-GlcCOONa (2.38 g).

A mixture of GlcA-1-P (0.428 g, 1.26 mmol) and Glc*_6_*-GlcCOONa (0.100 g, 0.084 mmol) in 0.2 mol/L sodium acetate buffer (6.0 mL, pH 6.2) was incubated in the presence of thermostable GP (30 U) at 50 °C for 48 h. The concentrated reaction solution was subjected to an anion exchange column (Amberlite IRA 400J Cl, Cl^−^ form, eluent: 1.0 mol/L aqueous acetic acid) and the fractions containing the products were collected and neutralized with 2.0 mol/L of aqueous Na_2_CO_3_. The resulting solution was dialyzed against water (molecular cut off: 1000) and concentrated at around 70 °C. The insoluble deactivated enzyme was removed using cotton plug filtration and the filtrate was lyophilized to obtain GlcA-Glc*_n_*-GlcCOONa (0.0221 g, 0.016 mmol) with a yield of 19.0%. ^1^H NMR (D_2_O) δ 3.49 (t, *J* = 9.6 Hz, GlcA-H4′′′), 3.57–4.03 (m, GlcA-H2,3,5, Glc-H2,3,4,5,6, GlcCOONa-H4,5,6), 4.11–4.13 (m, GlcCOONa-H2,3), 5.17 (m, Glc-H1′), 5.41 (m, Glc-H1″, GlcA-H1′′′).

### 3.3. Preparation of WSCS 

The fully deacetylated chitosan (1.0 g, 6.2 mmol) was dissolved in 2.0 wt% aqueous acetic acid (20 mL) by stirring at room temperature for 6 h. After adding 30 wt% aqueous H_2_O_2_ (5.0 mL) to the solution, the mixture was stirred at 50 °C for 4 h. After the resulting mixture was neutralized with 10 wt% aqueous NaOH, the residue was removed by centrifugation and the supernatant was dialyzed against water (molecular cut off: 1000). The precipitate produced during the dialysis was removed using centrifugation and the supernatant was lyophilized to obtain WSCS (0.0373 g, 2.3 mmol) with a yield of 37.3%. ^1^H NMR (D_2_O) δ 2.89 (br s, H2), 3.63–3.96 (m, H3,4,5,6), 4.87 (br s, H1). The viscosity average molecular weight (*M*_v_) of the resulting water-soluble chitosan was estimated according to the literature method [26] to be 14,000.

### 3.4. Preparation of Nanogels

The typical experimental procedure was as follows (run 1, NH_2_:COONa = 1:0.04). After a solution of GlcA-Glc*_n_*-GlcCOONa (5.2 mg, 3.72 μmol, COONa group; 7.50 μmol), 1-ethyl-3-(3-dimethylaminopropyl)carbodiimide hydrochloride (EDC, 1.43 mg, 7.44 μmol), and *N*-hydroxysuccinimide (NHS, 0.86 mg, 7.44 μmol) in water (0.20 mL) was left standing at room temperature for 1 h, it was mixed with an aqueous solution of WSCS (30 mg, 0.186 mmol/0.70 mL). The resulting mixture was then left standing at room temperature for 7 h to progress the condensation. The reaction mixture was poured into the methanol (30 mL) to precipitate the product. The precipitate was isolated via filtration and lyophilized to give the network polysaccharide (18.5 mg). ^1^H NMR (DCl/D_2_O) δ 3.09 (br s, GlcN-H2), 3.27–3.82 (m, GlcA-H2,3,4,5, Glc-H2,3,4,5,6, GlcCOOH-H2,3,4,5,6, GlcN-H3,4,5,6), 4.54 (m, Glc-H1β), 4.75, (m, GlcN-H1β), 4.87 (m, β(1→4)-linked GlcN-H1), 5.07 (m, Glc-H1α), 5.32 (m, GlcN-H1α).

### 3.5. Measurements

The IR spectra were recorded on a PerkinElmer Spectrum Two spectrometer (PerkinElmer Japan Co., Ltd., Kumamoto, Japan). The ^1^H NMR spectra were recorded on a JEOL ECX400 spectrometer (JEOL, Akishima, Tokyo, Japan). The viscosity ratees were measured with a Rheosol-G1000 rheometer (UBM, Kyoto, Japan). The DLS measurements were performed on a ELSZ-2000ZS zeta-potential and particle size analyzer (Otsuka Electronics Co., Ltd., Hirakata, Osaka, Japan). The SEM images were obtained using the Hitachi S-4100H electron microscope (Hitachi High-Technologies Corporation, Tokyo, Japan) by applying a 5 kV accelerating voltage. The TEM images were obtained using the JEOL JEM-3010 electron microscope (JEOL, Akishima, Tokyo, Japan). The TEM samples were prepared by drop-casting aqueous solutions on the carbon-coated copper grid and then staining them with the potassium phosphotungstate solution.

## 4. Conclusions

We report here on the fabrication of CS-based network polysaccharide nanogels by the chemical crosslinking of WSCS with GlcA-Glc*_n_*-COONa. The crosslinking was induced through the condensation of the amino groups in the WSCS with the carboxylate groups in the GlcA-Glc*_n_*-COONa in the presence of a condensing agent in water. The structure of the crosslinked products was confirmed using an IR analysis and ^1^H NMR measurements after dissolution via acid hydrolysis in DCl/D_2_O. The SEM, TEM, and DLS measurements suggested the formation of nanogels from the crosslinked products. Their size increased owing to crosslinking among larger numbers of CS chains with GlcA-Glc*_n_*-COONa, according to the COONa/NH_2_ feed ratio. Because the currently used CS-based nanogels are composed entirely of saccharide chains, they have potential practical applications as new polysaccharide-based 3D bionanomaterials. For example, the present nanogel materials will be employed as matrixes for delivery and release systems after encapsulating drugs. As the aim of the present study was to develop the method to fabricate nanogels based on network polysaccharide structures, further studies on the additional properties of the present materials, such as the biological properties, will also be reported in our forthcoming papers.

## Figures and Tables

**Table 1:** Preparation and size characterization of CS-based network polysaccharide nanogels ^(a)^.

Run	NH_2_:COONa	Glc/GlcN Unit Ratio ^(b)^	Particle Size by SEM (nm)	Standard Deviation by SEM	Particle Size by DLS (nm)
1	1:0.04	0.23	44	0.63	720.0
2	1:0.05	0.33	90	0.82	729.6
3	1:0.06	0.39	128	1.11	909.8
4	1:0.10	0.45	135	1.09	975.2
5	1:0.12	0.58	242	2.07	1366.8

^(a)^ The reaction was carried out at room temperature for 7 h at a feed ratio of COONa/EDC/NHS = 1:1:1. ^(b)^ Determined using the ^1^H NMR spectra after the acid hydrolysis of the products in DCl/D_2_O.

## Data Availability

Not applicable.

## Supplementary Materials

The following supporting information can be downloaded at: https://www.mdpi.com/article/10.3390/molecules27238384/s1. Figure S1: Synthesis of carboxylate-terminated maltooligosaccharides (GlcAGlc*_n_*-GlcCOONa). Figure S2: IR spectra of (a) water-soluble chitosan and (b) chitosan-based network polysaccharide (run 4). Figure S3: DLS profiles of chitosan-based network polysaccharides.
