# Supplementary material for: Fabrication of Chitosan-Based Network Polysaccharide Nanogels
Source: Molecules. 2022 Dec 1;27(23):8384. doi: 10.3390/molecules27238384 (PMC9740819; doi:10.3390/molecules27238384)
Supplement: Supplementary file 1 [file molecules-27-08384-s001.zip › molecules-2053401-supplementary.pdf]

Supporting Information

Fabrication of Chitosan-based Network Polysaccharide Nanogels

Aina Nakamichi and Jun-ichi Kadokawa*

Graduate School of Science and Engineering, Kagoshima University, 1-21-40 Korimoto, Kagoshima 890-0065, Japan

* Corresponding author: E-mail: kadokawa@eng.kagoshima-u.ac.jp

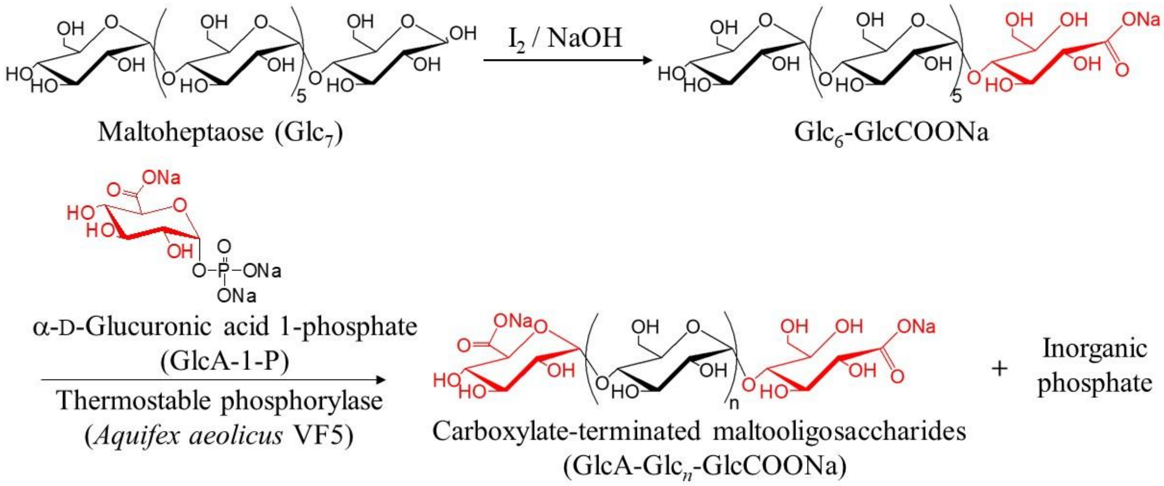

Figure S1. Synthesis of carboxylate-terminated maltooligosaccharides (GlcAGlc$_n$-GlcCOONa).

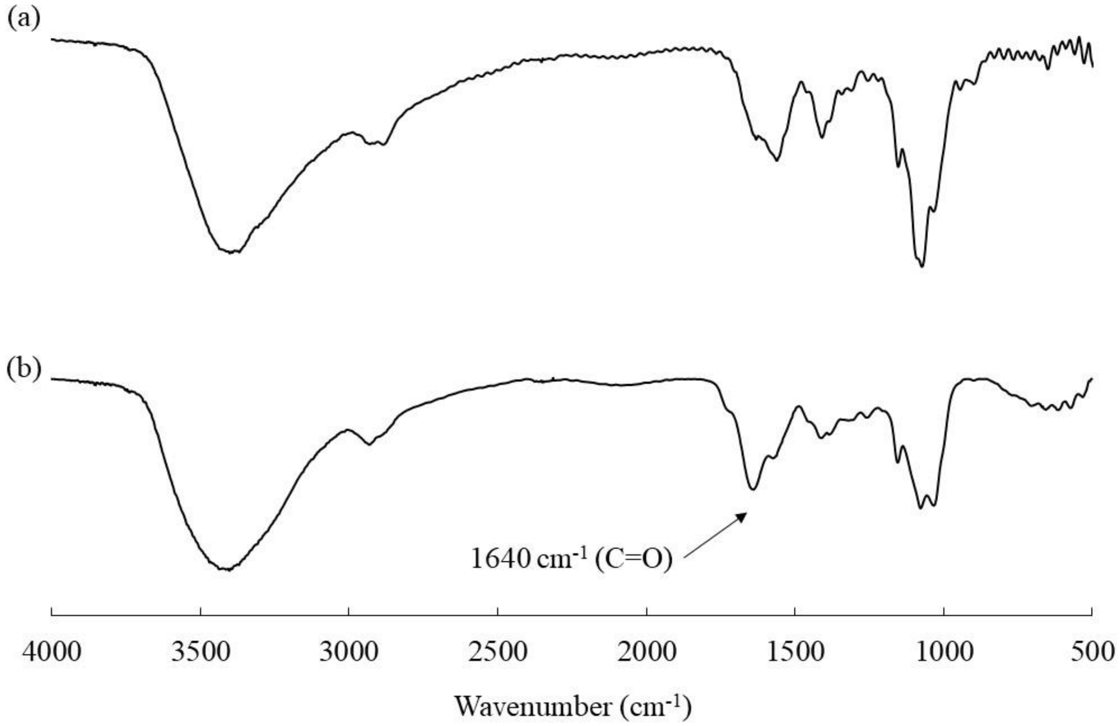

Figure S2. IR spectra of (a) water-soluble chitosan and (b) chitosan-based network polysaccharide (run 4).

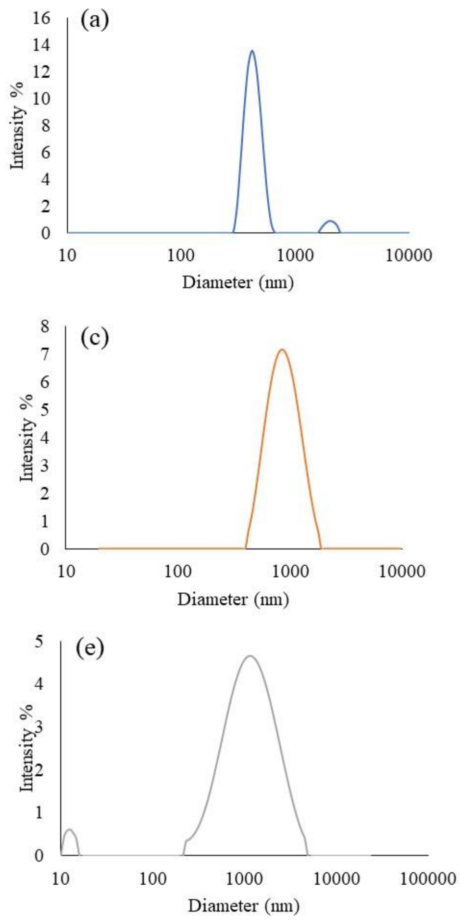

Figure S3. DLS profiles of chitosan-based network polysaccharides (a-e, runs 1-5).
